# Molecular identification and detection of virulence genes among *Pseudomonas aeruginosa* isolated from different infectious origins

**Published:** 2012-09

**Authors:** VS Nikbin, MM Aslani, Z Sharafi, M Hashemipour, F Shahcheraghi, GH Ebrahimipour

**Affiliations:** 1 Department of Microbiology, Pasteur Institute of Iran, Iran; 2 Department of Biology, Shahid Beheshti University, Iran

**Keywords:** *Pseudomonas aeruginosa*, ribotyping, virulence factors

## Abstract

**Background and Objectives:**

*Pseudomonas aeruginosa* possesses a variety of virulence factors that may contribute to its pathogenicity. The aim of this study was to evaluate *oprI*, *oprL* and *toxA* genes for PCR identification of clinical *P. aeruginosa*. In order to find out any relation between special virulence factors and special manifestation of *P. aeruginosa* infections, we detected virulence factors among these isolates by PCR. Ribotyping was used to evaluate the clonal relationship between strains with the same genetic patterns of the genes studied.

**Materials and Methods:**

In this study, 268 isolates of *P. aeruginosa* were recovered from burn, wound and pulmonary tract infections. The prevalence of *oprI*, *oprL*, *toxA*, *lasB*, *exoS* and *nan1* genes was determined by PCR. One hundred and four isolates were selected randomly to investigate clonal diversity of the isolates with ribotyping using *Sma*I.

**Results and Conclusions:**

All *P. aeruginosa* isolates in this study carried *oprI*, *oprL* and *lasB* genes. Difference between *exoS* prevalence in isolates from pulmonary tract and burn isolates was statistically significant. Prevalence of *nan1* and *tox*A gene was significantly higher in pulmonary tract and burn isolates, respectively. Ribotyping showed that most of the isolates (87%) belonged to clone A and B.

Detection of *oprI*, *oprL* and *toxA* genes by PCR is recommended for molecular identification of *P*. *aeruginosa*. Determination of different virulence genes of *P. aeruginosa* isolates suggests that they are associated with different levels of intrinsic virulence and pathogenicity. Ribotyping showed that strains with the same genetic patterns of the genes do not necessarily have similar ribotype patterns.

## INTRODUCTION

*Pseudomonas aeruginosa* is an opportunistic pathogen capable of infecting virtually all tissues. Pulmonary tract colonization with mucoid *P. aeruginosa* is a major cause of morbidity and mortality in patients with cystic fibrosis (1). *P. aeruginosa* infections in hospitals mainly affect the patients in intensive care units and those having catheterization, burn, and/or chronic illnesses (2).

Although conventional microbiological methods for identifying *P. aeruginosa* from clinical and environmental samples are reliable, they require several days to be completed. Rapid detection of isolates causing hospital infections is very important for consequent treatment decision of patients.

PCR has the potential for identifying microbial species rapidly by amplification of sequences unique to a particular organism (3). L and I lipoproteins are two outer membrane proteins of *P. aeruginosa* responsible for inherent resistance of *P. aeruginosa* to antibiotics and antiseptics. As these proteins are found only in this organism, they could be a reliable factor for rapid identification of *P. aeruginosa* in clinical samples (4–6).

*P. aeruginosa* also has a large number of virulence factors such as exotoxin A, exoenzyme S, elastase and sialidase which are tightly regulated by cell-to-cell signalling systems (7). Protein biosynthesis is inhibited by exotoxin A and virulence factor exoenzyme S is secreted by a type III section system (8, 9). A zinc metalloprotease called Las B has an elastolytic activity on lung tissue (10). The gene called *nan1* encodes a sialidase that is responsible for adherence to the respiratory tract (11).

In epidemiologic studies, dissemination of resistant and highly virulent pathogens is also the main problem worldwide. Because of highly conserved rRNA sequences among the eubacteria, chromosomal DNA restriction fragment length polymorphism of rRNA genes (ribotyping) is a powerful approach to discriminate strains, between and within species (12).

In this study, we examined rapid identification of *P. aeruginosa* isolated from pulmonary tract, wound and burn samples based on PCR amplification of I lipoprotein (*oprI*) for detection of genus and L lipoprotein (*oprL*) for detection of species of this organism. The *toxA* gene was also examined to evaluate molecular detection of the isolates by this factor. Furthermore, in order to find any relation between special virulence factors and special manifestation of *P. aeruginosa* infections, we detected *exoS*, *nan1*, *lasB* virulence factors among these isolates by PCR. Ribotyping was also used to evaluate the clonal relationship between strains with the same genetic patterns of the genes studied in this research.

## MATERIALS AND MTHODS

### Bacterial strains and identification test

Totally 268 *P. aeruginosa* isolates including 100 strains recovered from burn, 50 from wound and 118 from pulmonary tract infections were obtained from patients admitted in four hospitals in Tehran, Iran. Each strain was identified on the basis of colony morphology and conventional biochemical testes (13).

### Preparation of bacterial DNA

All isolates were inoculated aerobically on tryptose soy broth for 18–24 hour at 37° C. Bacterial DNA extraction was performed using phenol-chloroform method as previously described (14).

### Detection of virulence genes by PCR

PCR amplifications of the *oprI, oprL, toxA, exoS, nan1* and *lasB* genes were performed in 25 µl reaction mixture containing 0.5 µl of dNTPs (10 mM), 0.5 µl of each primer (10 pmol), 1.5 µl MgCl_2_ (25 mM), 0.2 µl *Taq* DNA polymerase (5 U/µl) (Fermentas, Lithuania) (3, 4, 15). Each gene was amplified separately. *Pseudomonas aeruginosa* ATCC 27853 and *E. coli* ATCC 25922 were used as positive and negative control respectively. PCR products were visualized by electrophoresis using a 1% agarose gel stained with ethidium bromide.

### Ribotyping

Ribotyping was performed as described previously (16). In brief, the extracted DNA from *P. aeruginosa* strains was cleaved by *Sma*I restriction endonuclease (Fermentas, Lithuania). The fragments were separated by electrophoresis and then transferred to nylon membrane by vacuum blotter (Bio-Rad Laboratories, Hercules, CA). Hybridization was performed by probes labeled with digoxigenenin. The membrane was then visualized by NBT (nitroblue tetrazolium) and BCIP (5-bromo-4-chloro-3-indolyl phosphate).

### Statistical method

The distribution of virulence genes with respect to strain origin was compared using the Chi square test.

## RESULTS

The *oprI* and *oprL* genes were detected in all of 268 *P. aeruginosa* isolates collected. However, presence of *toxA* gene in clinical samples was different. According to Table 1 the presence of *toxA* gene in isolates from burn was significantly higher than pulmonary tract (*P* < 0.05).

**Table 1:** Prevalence of *toxA*, *exoS* and *nan1* among *P. aeruginosa* obtained from various sources.

	1	2	3	
		
Virulence gene	Wound (%) (n**=** 50)	Burn (%) (n = 100)	Pulmonary tract (%) (n = 118)	P value (Chi square test)
*toxA*+	(45) 90	(97) 97	101 (85.6)	*0.004 = (2,3)
*exos*+	31 (62)	(67) 67	56 (47.4)	*0.004 = (2,3)
*nan1*+	15 (30)	(4) 4	55 (46.6)	(1,2) = 0.000 = (1,3)* *0.000 = (2,3)* 0.04

Our results showed that all tested isolates harbored *lasB* gene. However, difference between *exoS* prevalence in isolates from pulmonary tract and burn isolates was statistically significant (*P* < 0.05). The *nan1* gene, other virulence factor studied in this research, was found in 55 (46.6%) of 118 isolates from pulmonary tract, 15 (30%) of 50 from wound and 4 (4%) of 100 from burn specimens. The prevalence of *nan1* gene was significantly higher in isolates of pulmonary tract than burn specimens (*P* < 0.05). There was a borderline significant difference in the prevalence of *nanI* gene among the isolates from pulmonary tract and wound infections. Furthermore, the prevalence of *nan1* among wound isolates was significantly higher than burn isolates (*P* < 0.05) (Table 1).

The isolates were divided into 8 genetic groups (I-VIII) based on the presence of six genes amplified by PCR (Table 2).

**Table 2:** Various genetic groups of *P. aeruginosa* isolates according to the presence of virulence genes. *oprI*, *oprL* and *lasB* occurred in all isolates.

Isolate	Wound	Burn	Pulmonary tract	Total
	
Genetic group				
I	14	3	16	33
II	1	1	31	33
III	17	36	33	86
IV	13	57	21	91
V	2	2	2	6
VI	3	1	7	11
VII	0	0	5	5
VIII	0	0	3	3
Total	50	100	118	268

group I: presence *toxA*, *exoS* and *nan1*; group II: presence *toxA* and *nan1*; group III: presence *toxA* and *exoS*; group IV: presence *toxA*; group V: presence *exoS*; group VI: nonexistence *toxA*, *exoS* and *nan1*; group VII: presence *exoS* and *nan1*; group VIII: presence *nan1*.

**Fig. 1:**
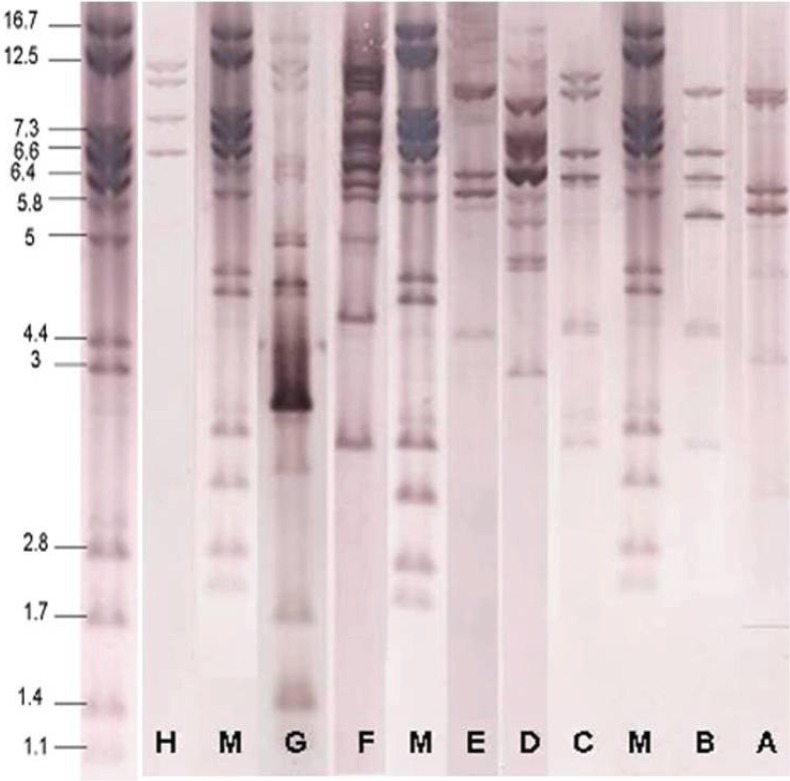
Ribotyping of *P. aeruginosa*. M ribotype is related to *Citrobacter koseri* CIP 105177 (Grimont 32) that selected as marker.

Of 8 genetic groups obtained from PCR results, 104 isolates were randomly selected to investigate clonal diversity of the isolates by ribotyping. Ribotyping patterns of the isolates compared by visual inspection. Ribotyping analysis generated 8 distinct patterns (A-H) (Fig. 1). These isolates were distributed in ribotypes A (53 isolates), B (28 isolates), C (5 isolates), D (4 isolates), E (1 isolate), F (1 isolate), G (1 isolate) and H (1 isolate) patterns (Table 3).

**Table 3:** Numbers and ribotype patterns for genetic groups of *P. aeruginosa* isolates.

Genetic group	I	II	III	IV	V	VI	Total
	
Isolate							
Wound	11 (8A,3B)	1 (1B)	11 (6A,4B,1E)	13 (2A,10B,1C)	1 (1D)	3 (2C,1F)	40 (16 A,18B,3C,1D,1E,1F)
Burn	2 (2A)	0	19 (17A,2B)	10 (7A,3B)	0	3 (1G,1A,1H)	34 (27A,5B,1G,1H)
Pulmonary tract	4 (4A)	12 (4A,6B,2C)	4 (4B)	4 (1A, 2B,1D)	1 (1B)	5 (1A,2B,2D)	30 (10A,15B,2C,3D)
Total	17 (14A,3B)	13 (4A, 7B,2C)	34 (23A,10B,1E)	27 (10A,15B,1C,1D)	2 (1D,1B)	11 (2A,2B,2C,2D,1F,1G,1H)	104 (53A,38B,5C,4D,1E,1F,1G,1H)

## DISCUSSION

Identification of *P*. *aeruginosa* has traditionally relied on phenotypic methods. This still is the most accurate standard when dealing with typical isolates of *P*. *aeruginosa*. In cystic fibrosis (CF) patients, *P*. *aeruginosa* isolates display unusual phenotypic reactions (17). Moreover, biochemical testing takes long time to perform and requires extensive hands-on work by the technologist, both for setup and for ongoing evaluation. Molecular methods have been reported to be superior to the phenotypic methods for identification of *P*. *aeruginosa* (17).

De Vos *et al*. (1997) by designing a multiplex PCR assay based on *oprI* and *oprL* genes for molecular detection of *P. aeruginosa* showed that the specificity and sensitivity of the PCR assay were 74 and 100%, respectively (4). Lavenir *et al*. also noted that all of *P. aeruginosa* strains contained the *oprI* and *oprL* genes (sensitivity = 100%, specificity = 80%). Similarly in this study, all of the 268 isolates were remarkably positive for both *oprI* and *oprL* genes (18).

According to these studies, detection of *P*. *aeruginosa* by PCR of *oprI* and *oprL* genes has a high sensitivity but a low specificity. The reason of low specificity of *oprI* and *oprL* genes is that, although the entire genome of *P*. *aeruginosa* has been sequenced, the genomes of its closest relatives have not. Thus, presence of false positive results among other species of bacteria during PCR assay of *oprI* and *oprL* genes indicate that they may have some similar sequences to *oprI* and *oprL* genes in their genomes (17). Consequently, use of only single gene target for molecular identification of *P*. *aeruginosa* potentially suffers from the same polymorphisms that complicate biochemical identification of this organism.

Khan and Cerniglia also developed a PCR procedure to detect *P*. *aeruginosa* by amplifying the *toxA* gene (3). They reported that of 130 tested *P*. *aeruginosa* isolates, 125 (96%) contained the *toxA* gene (sensitivity = 96%), whereas other species of bacteria did not yield any positive results (specificity = 100%). Qin *et al*. and Lavenir *et al*. also reported similar results. These studies indicate that, unlike *oprI* and *oprL* genes, detection of *P*. *aeruginosa* by PCR of *toxA* gene has a high specificity but a low sensitivity (17, 18). In this study our results also showed that 243 (90.7%) of 268 isolates harbored *toxA* gene.

The *ptxR* gene, expression enhancer of *toxA* gene, was only detected in *P*. *aeruginosa* isolates; whereas other species of *Pseudomonas* did not yield any positive results (19). Low sensitivity with *toxA* PCR screening is due to the fact that some isolates of *P*. *aeruginosa* do not carry this gene naturally.

Pathogenicity of *P*. *aeruginosa* is clearly multifactorial. LasB is one of the most important proteases of *P*. *aeruginosa* (20). In this study all isolates examined harbored *lasB* gene. This finding is in agreement with previous reports (20, 21). Mutation of *lasB* gene reduces markedly *P*. *aeruginosa* invasion (22). Prevalence of the *lasB* gene in all the environmental and clinical isolates implies the importance of LasB factor to survival of *P*. *aeruginosa* in various settings.

*P*. *aeruginosa* isolates generally express cytotoxicity or invasion phenotypes which is correlated with presence of *exoU* (encoding exotoxin U) or *exoS* (encoding exotoxin S) respectively (23). In our study difference between *exoS* prevalence in the isolates from pulmonary tract and burn infections was statistically significant (*P* < 0.05) (Table 1).

The proportion of isolates from pulmonary tract infections that exhibited *exoS* (47.4%) in this study was lower than that previously reported (20, 15, 24, 25). The conflicting results of these studies may be due to differences in the number of clinical isolates from different sites or due to the isolates from patients with different clinical and physiological conditions (20, 24)

About the *nan1* gene, the other virulence factor studied in this research, we found that the prevalence of *nan1* was significantly higher in isolates from pulmonary tract than isolates from burn (*P* < 0.05). Furthermore, the prevalence of *nan1* among the isolates from wound was significantly higher than burn (*P* < 0.05). Similar to our results, Lanotte *et al*. reported that 7 (41.2%) of 17 wound isolates and 12 (48%) of 25 pulmonary tract isolates contained *nan1* gene. This gene has probably A role in CF pulmonary disease evolution as previously described (15). The low prevalence of this factor among isolates from burn infections may show that the role of this gene in the burn infections is less important than wound and pulmonary tract infections.

The differences in the distributions of virulence factor genes in the populations strengthen the probability that some *P*. *aeruginosa* strains are better adapted to the specific conditions found in specific infectious sites (15).

The isolates in this study were divided into 8 genetic groups (I-VIII) based on presence of the investigated virulence genes in the isolates. There was no correlation between clinical origin of *P*. *aeruginosa* isolates and their distribution in the 8 genetic groups (Table 2).

Although ribotyping is slightly less discriminatory than pulsed field gel electrophoresis (PFGE) (26), the high rate of interlaboratory reproducibility and the speed of the generation of results make this method a valuable approach for characterization of clinical bacteria (12). Ribotypoing demonstrated 8 distinct ribotype patterns (A-H). Fifty-three (51%) and 38 (36%) isolates belonged to ribotype pattern A and B, respectively. Indeed, most of the isolates (87%) belonged to ribotype pattern A and B. There was no significant meaningful correlation between genetic groups and ribotype patterns.

In conclusion, it seems that simultaneous use of *oprI*, *oprL* and *toxA* genes provides more confident detection of *P. aeruginosa* by PCR. Determination of different virulence genes of *P. aeruginosa* isolates suggests that they are associated with different levels of intrinsic virulence and pathogenicity. This may have different consequence on the outcome of infections. Significant correlations between some virulence genes and source of infections obtained in this research indicates that more further studies is required for finding out the actual role of these genes in different clinical infectious caused by *P. aeruginosa*. Ribotyping showed that strains with similar virulence genes do not necessarily have similar ribotype patterns. However, clonal spread of highly virulent isolates of *P. aeruginosa* within hospitals needs to apply additional precautions in clinical settings.
